# Rectal ectopic pregnancy after in vitro fertilization and embryo transfer: A case report

**DOI:** 10.1097/MD.0000000000031676

**Published:** 2022-11-25

**Authors:** Chujun Wang, Yipin Xiong, Fengzhen Liu, Lijuan Qiu, Chun-Quan Zhang

**Affiliations:** a Department of Ultrasound, The Second Affiliated Hospital of Nanchang University, Nanchang, China.

**Keywords:** abdominal pregnancy, case report, embryo transfer, laparoscopic surgery, ultrasonography

## Abstract

Rectal ectopic pregnancy is an extremely rare abdominal pregnancy. This article presents a female underwent an unsuccessful in vitro fertilization which was misdiagnosed by serum beta-human chorionic gonadotropin (β-hCG) test and transvaginal ultrasonography. Twenty days later, a ruptured rectal ectopic pregnancy was confirmed by laparoscopy then the gestational tissue removed successfully.

**Patient concerns:**

A 32-year-old Chinese female was admitted to our hospital with complaining of symptoms, like gradual worsening of lower abdominal pain and dysuria. The abdominal ultrasonography revealed a sac-like mass in the posterior area to the uterus and a moderate amount of free fluid in the pelvic cavity. Forty days ago, she underwent a frozen embryo transfer. Twenty days ago, her serum β-hCG level was <5 mIU/mL and neither intrauterine nor ectopic pregnancy was detected by transvaginal ultrasonography. Then the procedure was thought to have resulted in biochemical pregnancy failure.

**Diagnosis:**

The primary rectal ectopic pregnancy.

**Interventions:**

The mass was removed laparoscopic surgery.

**Outcomes:**

The patient recovered well.

**Lessons:**

When the history of in vitro fertilization combined with an inappropriate rise of serum β-hCG and no visible evidence of an intra-uterine pregnancy, physicians should consider the possibility of abdominal pregnancy. Early diagnosis of abdominal pregnancy can effectively save the life of the pregnant woman.

## 1. Introduction

Ectopic pregnancy is a common gynecological acute abdomen and ruptured ectopic pregnancy accounts for 2.7% of pregnancy-related deaths.^[1]^ With the development of assisted reproductive technology (ART), the incidence of ectopic pregnancy after in vitro fertilization (IVF) is 3.4%, which is significantly higher than that in vivo conception.^[2,3]^ Abdominal pregnancy is a rare kind of ectopic pregnancy, and it can be suspected based upon an elevated serum β-human chorionic gonadotropin (β-hCG) level in combination with ultrasound findings showing absence of an intrauterine gestational sac and a wide mobility similar to fluctuation of the sac.^[4]^ Unlike the positive initial serum β-hCG of the usual abdominal pregnancy following IVF, this case report a ruptured rectal ectopic pregnancy with an inappropriate rise of serum β-hCG.

## 2. Case report

A married, nulliparous, 32-year-old woman was referred to the local fertility center for IVF treatment. Her husband’s semen analysis was normal. In her past medical history, she underwent hysterosalpingography showing obstruction in the right fallopian tube. On May 31st, the patient underwent IVF and day 6 single frozen embryo transfer. After a menstrual period had occurred 11 days later, she presented to the center. Her serum β-hCG level was 2.31 mIU/mL and no dominant follicle development was identified by transvaginal ultrasonography (TVS). Thus, the procedure was thought to have resulted in biochemical pregnancy failure. Fortunately, the follow-up checks did not make regularly.

**Figure 1.**
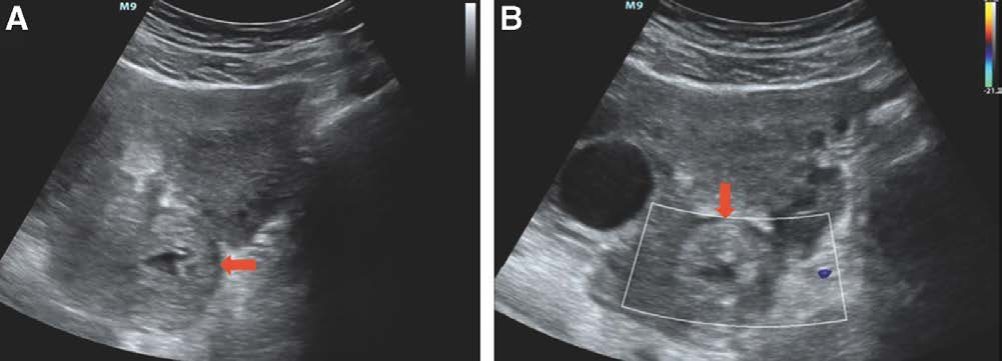
Abdominal ultrasound reveals a gestational sac with thick echogenic margins in the posterior to the uterus. (A) No signals of blood flow is shown within the gestational sac by color Doppler flow. (B).

On July 10th, she was admitted to our hospital with complaining of symptoms, like gradual worsening of lower abdominal pain, dysuria, diarrhea and rectal pressure lasting for 1 day. And she denied sexual behavior for nearly a month. Physical examination revealed muscle tightness in the lower abdomen. Blood pressure was 115/77 mm Hg and pulse rate was 89 beats per minute. Hemoglobin was normal at 112 g/L and hematocrit was lower than normal at 32.5%. Abdominal ultrasonography showed an endometrial thickness lining of 5mm with no intrauterine gestational sac identified and a moderate amount of free fluid in the pelvic cavity. A sac-like mass with a size of 54 mm × 24 mm was found in the posterior area to the uterus (Fig. 1A). Color Doppler ultrasonography indicated no signals of blood flow in the mass (Fig. 1B). Furthermore, anechoic mass measuring 33 mm × 18 mm was found in the right ovary and the serum β-hCG serum level was detected to be 12451.6 mIU/mL. Given the lack of visible intrauterine pregnancy, pelvic free fluid, positive β-hCG, history of IVF, and lower abdominal pain, there was concern for a ruptured ectopic pregnancy.

**Figure 2.**
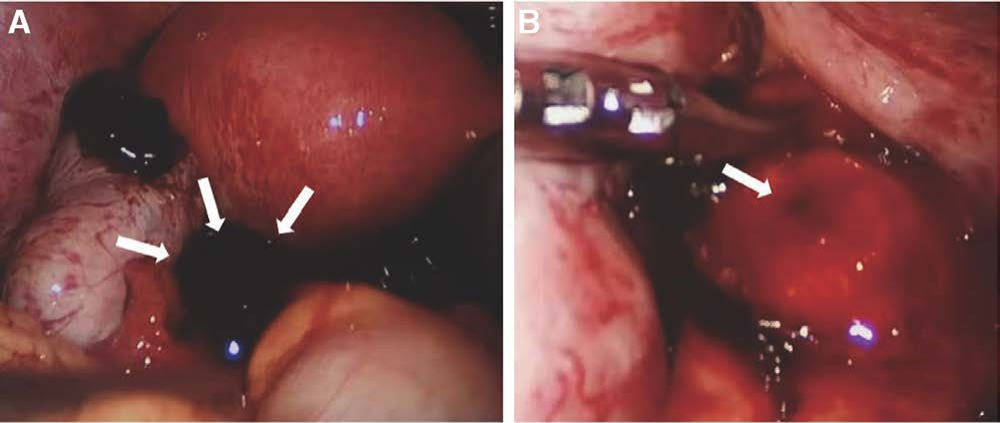
The bleeding lesion in the pouch of Douglas (white arrow). (A) A mucosal laceration on the anterior surface of rectum. (B).

The patient underwent laparoscopic exploration and surgical treatment. After suctioning of blood clots and hemoperitoneum (about 1000 mL), a ruptured mass was found in the pouch of Douglas (Fig. 2A). And an approximately 25-mm actively bleeding mucosal laceration was identified on the anterior surface of rectum and considered as an implanting location of gestational tissue (Fig. 2B). In addition, a 40-mm cyst and a 8-mm cyst were seen in the right ovary and right fallopian tube, respectively. The uterus, left ovary and left fallopian tube appeared normal. Finally, the mass and the cysts were dissected completely and there was no complications (e.g., rapid pulse, fall of blood pressure) of the surgery.

**Figure 3.**
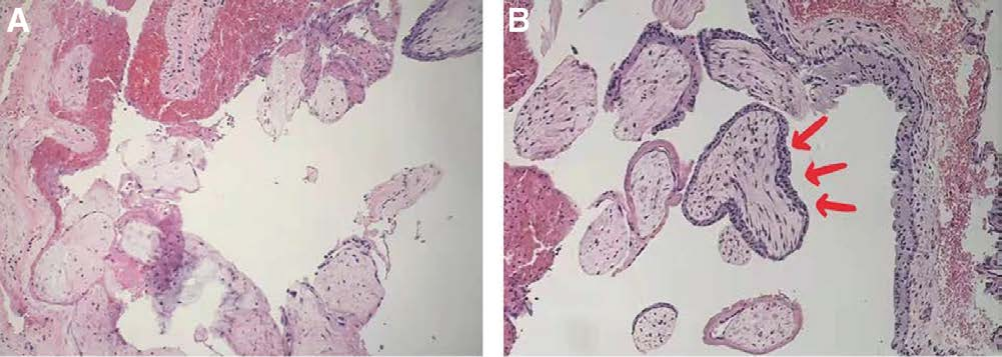
Histopathologic examination of the ectopic hemorrhagic tissue. Hematoxylin and eosin stain (A,B) of the hemorrhagic mass showing chorionic villi (red arrow) confirming an ectopic pregnancy.

The histopathological examination revealed chorionic villi within the hemorrhagic mass, confirming the diagnosis of an ectopic pregnancy (Fig. 3). Her serum β-hCG levels declined to 308 mIU/mL and hematocrit increased to 52.6% on post-operative day 6, then the patient was discharged. Upon outpatient follow-up on post-operative day 25, her serum β-hCG level was undetectable and repeat TVS showed there were no obvious abnormalities in the uterus and bilateral adnexal areas. And the patient has provided informed consent for publication of the case.

## 3. Discussion

Abdominal pregnancy is an extremely rare and aggressive type of ectopic pregnancy and the incidence ranges from 0.9% to 1.4%.^[5]^ It refers to pregnancy located outside the fallopian tubes, ovaries, and broad ligament and implanted in the abdominal cavity.^[6]^ An increasing number of studies have shown IVF is one of the risk factors leading to abdominal pregnancy.^[7–9]^ Although a majority of pregnancy could be visualized on TVS, ultrasonography alone is rarely used because yolk sac do not develop to this stage and exceedingly rare abdominal pregnancy may not identified.^[10]^ Thus, it is important to monitor the serum β-hCG level. Because the date of conception with IVF is precise, a single measurement of serum β-hCG at 12 to 18 days following embryo transfer has been established as a reliable early indicator of pregnancy.^[11]^ And biochemical pregnancy failure is usually defined as a serum β-hCG < 5 mIU/mL 14 days after embryo transfer without a visible gestational sac by TVS.^[12]^ Here, we report a ruptured rectal ectopic pregnancy which was thought to a biochemical pregnancy failure at the first postoperative visit.

Different from rectal ectopic pregnancies following IVF that have been reported, in this case, the initial serum β-hCG level was 2.31 mIU/mL and neither intrauterine nor ectopic pregnancy was found by TVS.^[13,14]^ And there were 3 similar abdominal pregnancies from 2000 to 2020 (Table 1).^[9,15,16]^ These cases have common characteristics: the history of IVF failure and the delayed serum β-hCG rise. It is suspected that a small number of embryos may lead slower proliferative activity of the trophoblast tissue and the abnormal implantation site may cause the poor absorption of β-hCG into the blood circulation.^[13]^ Although serum β-hCG is most important indicator for the diagnosis of abdominal pregnancy, the possibility of occult abdominal pregnancy should not be excluded when the first serum β-hCG level was < 5 mIU/mL. The best treatment of abdominal pregnancy is surgical removal of gestational tissue as soon as possible, and surgical outcomes are mostly satisfactory.

**Table 1:** Review of the cases of abdominal pregnancy with initial serum β-hCG level <5 mIU/mL after IVF.

	Yanaihara et al^[9]^	Irani et al^[15]^	Li et al^[16]^	Our case
Age	37	36	26	32
Parity	G0P0	G0P0	G0P0	G1P0
ART reason	5-yr history of infertility	1.5-yr history of infertility	3-yr history of infertility	Primary infertility
Fresh/Frozen embryo	Frozen	Fresh	Frozen	Frozen
Number of embryo	1	1	2	1
Inital serum β-hCG level	3.4 mIU/mL (9 d PT)	Undetectable (9 d PT)	2.59 mIU/mL (14 d PT)	2.31 mIU/mL (11 d PT)
Stage at diagnosis	46 d PT	28 d PT	33 d PT	40 d PT
β-hCG level	The positive urinary pregnancy test	1,958 mIU/mL	4,103 mIU/mL	12,451.6 mIU/mL
Ultrasonographic findings	A gestational sac containing a fetus near the pouch of Douglas	A moderate amount of free fluid with no pregnancy identified	A bloody fluid; A gestational sac–like echo near the posterior wall of uterus	A moderate free fluid; A sac-like mass in the posterior area to the uterus
Location	The pouch of Douglas	The anterior bladder flap	The posterior wall of uterus	The anterior wall of rectum
Rupture?	No	Yes/1000 mL	Yes/400 mL	Yes/1000 mL
Surgery	Laparoscopy	Laparoscopy	Laparoscopy	Laparoscopy

β-hCG = β-human chorionic gonadotropin, ART = assisted reproductive technology, IVF = in-vitro fertilization, PT = post-embryo transfer.

## 4. Conclusion

Abdominal pregnancy is an extremely rare and aggressive type of ectopic pregnancy. If the initial serum β-hCG level was < 5 mIU/mL following IVF, occult abdominal pregnancy could not be excluded. When the history of IVF combined with an inappropriate rise of serum β-hCG and no visible evidence of an intra-uterine pregnancy, physicians should consider the possibility of abdominal pregnancy. To reduce the mortality of the disease, paying attention to repeat examination of the serum β-hCG level and TVS is effectively choice.

## Author contributions

**Conceptualization:** Chujun Wang, Fengzhen Liu, Chun-Quan Zhang.

**Data curation:** Yipin Xiong, Lijuan Qiu.

**Project administration:** Chun-Quan Zhang.

**Resources:** Yipin Xiong, Chun-Quan Zhang.

**Writing – original draft:** Chujun Wang.

**Writing – review & editing:** Chujun Wang.
